# *Zuo Gui Wan* Alters Expression of Energy Metabolism Genes and Prevents Cell Death in High-Glucose Loaded Mouse Embryos

**DOI:** 10.1155/2018/2409471

**Published:** 2018-06-25

**Authors:** Qi Liang, Zhipeng Qu, Yu Liang, QianJin Feng, Xin Niu, Temaka Bai, Yingli Wang, Qiang Song, David L. Adelson

**Affiliations:** ^1^Shanxi University of Chinese Medicine, Taiyuan 030619, China; ^2^School of Biological Sciences, University of Adelaide, Adelaide, SA 5005, Australia; ^3^Beijing University of Chinese Medicine, Beijing 100029, China

## Abstract

**Background:**

* Zuo Gui Wan* (ZGW) is a classic formula in traditional chinese medicine (TCM). Previous studies have shown that it is beneficial for impaired glucose tolerance (IGT) of adults and the offspring as well. This study aimed to understand the molecular mechanisms of the efficacy of ZGW on IGT.

**Methods:**

We used high-glucose loaded 2-cell stage mouse embryos as a model and took advantage of single-cell RNA sequencing technology to analyze the transcriptome of the model with or without ZGW. Differential gene expression analysis was performed with DESeq2.

**Results:**

High glucose can downregulate genes in the ribosome pathway, while ZGW can reverse this inhibition and as a result prevent embryo cell death caused by high glucose. Furthermore, high glucose can affect sugar metabolism and influence mitochondrial function, but ZGW can promote sugar metabolism via the tricarboxylic acid cycle mainly through upregulating the genes in the respiratory chain and oxidative phosphorylation.

**Conclusions:**

ZGW had a protective effect on embryonic cell death caused by glucose loading. The reversion of inhibition of ribosome pathway and regulation of mitochondrial energy metabolism are main effects of ZGW on high-glucose loaded embryos. This research not only revealed the global gene regulation changes of high glucose affecting 2-cell stage embryos but also provided insight into the potential molecular mechanisms of ZGW on the IGT model.

## 1. Background

Impaired glucose tolerance (IGT) is a sugar metabolism disorder between normal glucose tolerance (NGT) and diabetes mellitus [1]. It is estimated that there are 308 million people with IGT in the world [2], many more than those diagnosed with diabetes. People with IGT can progress to DM and are predisposed to cardiocerebrovascular disease [3–8], microvascular disease [9], lipid metabolism disorders [10], and chronic kidney disease [11, 12]. Therefore, it is becoming more and more important to understand how to prevent IGT. Currently, drugs such as Metformin, Acarbose, and Rosiglitazone are used to treat IGT and can postpone the occurrence of diabetes; however, they cannot prevent corresponding complications [13–18]. Many reports have shown that TCM might be one of the resources to develop new methods for preventing IGT [19–25].

In a previous report, three different Chinese formulas,* Zuo Gui Wan *(ZGW),* You Gui Wan *(YGW), and* Ba Zhen Tang* (BZD), were separately used to treat pregnant Wistar rats. After drugs were administered for three weeks, offspring from different groups were then fed with a high-fat diet for 12 weeks. Many indices, such as fasting blood glucose (FBG), 2-hour blood glucose (2hBG), blood lipid, fasting serum insulin (FINS), and leptin and adiponectin (APN), were measured. Results showed that rats on a high-fat diet developed IGT with abnormal blood lipid, insulin resistance, leptin resistance, and fatty liver. However, ZGW could prevent IGT in these offspring. Compared to YGW and BZD, which only reversed part of above indexes, ZGW was the most effective formula [26]. Another recent report has also found that giving ZGW to Gestational Diabetes Mellitus (GDM) rats can have a preventive effect on the IGT of offspring induced by a high-fat and high-sugar diet [27]. ZGW is a classic traditional Chinese medicine (TCM) formula with extract from 8 traditional Chinese medicines, which are* Rehmannia glutinosa* (Shu Di Huang),* Cuscuta chinensis* (Tu Si Zi),* Cornus officinalis* (Shan Zhu Yu),* Lycium barbarum* (Gou Qi Zi),* Dioscorea opposita* (Shan Yao),* Cyathula officinalis* (Chuan Niu Xi),* Cervi cornus Colla *(Lu Jiao Jiao), and* Chinemys reevesii* (Gui Ban Jiao). 12 main metabolites were detected in ZGW rat serum with UPLC/MS: *β*-D-ribofuranuronic acid methyl ester triacetate, 5-hydroxymethyl-2-furfural glucuronide, dihydro-5-hydroxymethyl-2-furfural glucuronide, 8-epiloganic acid, loganic acid, morroniside, coumaric acid, loganin, sweroside, 3-hydroxy-2,6,6-trimethyl-1-cyclohexene-1-carboxylic acid, kaempferol-3-glucuronide, and cuscutamine. Of these, morroniside and loganin could regulate rat mesangial cell growth by reducing oxidative stress and could be used at the early stages of diabetic nephropathy [28]. Studies have shown that dietary kaempferol may reduce the risk of chronic diseases, especially cancers, by augmenting antioxidants to combat free radicals [29].

Previous studies have shown that ZGW is an effective treatment in the IGT rat model, and it is beneficial not only to the mother but also to the offspring [26, 27, 30]. But the molecular mechanisms of ZGW on IGT, particularly at the transcriptome level, are still unclear. Therefore, in this study, we used mouse embryos loaded with high glucose as an IGT model to study the effect of ZGW. By analyzing the transcriptome of our IGT model treated with ZGW, we identified ribosome pathway and oxidative phosphorylation as the potential target molecular pathways of ZGW on IGT. A list of potential response genes to ZGW on IGT was also identified, and these genes provide a good resource for further functional studies.

## 2. Methods

All chemicals used in this study were purchased from Sigma-Aldrich Corporation (St. Louis, MO, USA) unless otherwise indicated. The Institutional Animal Care and Use Committee at the China Agricultural University (Beijing, China) approved the protocols used in this study.

### 2.1. Preparation of ZGW

ZGW is a classic formula in TCM and includes the extract from 8 traditional Chinese medicines. First, these 8 medicines were immersed in 800 ml water at 50°C and then decocted for 1.5 hours and filtered. The decoction and filtration were repeated two times. The filtrates were combined and concentrated to 1 g/ml crude drug.

### 2.2. ZGW Serum Preparation

Rats from National Institutes for Food and Drug Control, China (license number: SCXK-(JING) 2009- 0017), were fed with 20 g/kg/d ZGW for 7 days. Blood was collected directly from their hearts and incubated at 4°C for 30 min, followed by centrifugation at 4000 rpm for 15 min at 4°C. The serum, denoted as ZGW-containing rat serum, was collected immediately and stored at −75°C before use. The concentration of ZGW rat serum used in this research was 0.01% v/v ZGW.

### 2.3. Super Ovulation

For timed pregnancy, pregnant mare's serum gonadotropin (PMSG) (5 IU) was intraperitoneally injected into ICR female mice aged 6 to 8 weeks. Next, human chorionic gonadotropin (hCG) (5 IU) was intraperitoneally injected after 48 h; on the evening of hCG injection, male mice and the female mice (2 : 1) were housed together in a cage for one night. The next morning, females were checked for a vaginal plug to determine if they were pregnant.

### 2.4. Drug Administration and Grouping

Pregnant mice were cervically dislocated and zygotes were washed out from the vagina. All zygotes were randomly assigned to three groups: control group, model group, and drug group. Each group contained 9 zygotes. Zygotes in different groups were cultured with media as follows: control group with cell-culture medium, model group with cell culture medium supplemented with high glucose 15.6 mmol/L, and drug group similar to the model group but with the addition of rat serum containing 0.01% v/v ZGW.

### 2.5. Determination of Blastocyst Embryo Cell Number

Zygotes in the three groups were cultured* in vitro* for five days (blastocyst stage) and then incubated separately in M2 medium [NaCl (5.533 g/L), KCl (0.356 g/L), CaCl_2_·2H_2_O (0.252 g/L), KH_2_PO_4_ (0.162 g/L), MgSO_4_·7H_2_O (0.293 g/L), NaHCO_3_ (0.349 g/L), Hepes (4.969 g/L), sodium lactate (2.610 g/L), sodium pyruvate (0.036 g/L), glucose (1.000 g/L), BSA (4.000 g/L), penicillin (0.060 g/L), and streptomycin (0.050 g/L)] containing Hoechst 33342 (10 *μ*g/mL) for 15 minutes at 37°C. After washing three times with M2 medium, blastocysts from the three groups were separately mounted on microscope slides and examined on an epifluorescence microscope to count the number of embryo cell nuclei.

### 2.6. Sample Preparation for Single-Cell RNA Sequencing

Zygotes from the three groups were cultured in vitro to 2-cell stage, and three zygotes were randomly selected as a sample from each group and stored in liquid nitrogen. Each group was replicated three times for biological replication. Zygotes were resuspended in freshly prepared lysis buffer [total volume 100 *μ*l, containing 93 *μ*l nuclease-free water, 2 *μ*l Triton X-100, and 5 *μ*l RNaseOUT (20 U/*μ*l)]; each zygote was lysed with a micropipette to yield more than 7 *μ*l of lysate and the lysate stored at −80°C.

### 2.7. Single-Cell RNA Sequencing and Bioinformatic Analysis

RNA amplification was performed according to SMARTer Ultra Low Input RNA for Illumina Kit (Clontech Laboratories). Quantity and quality of amplified cDNAs were measured with Qubit and Agilent Bioanalyzer 2100 (Agilent Technologies, Santa Clara, CA, USA). RNA sequencing was performed using Illumina HiSeq 2500 (Shanghai Biotechnology Corporation, Shanghai, China). Adaptors and low-quality sequences from raw reads were filtered using Trim Galore! with the following parameter: stringency 6. Trimmed reads were mapped to the mouse reference genome (mm10) using STAR v2.5 with the following parameters: out Filter Mismatch Nover *L* max 0.05 and seed Search Start *L* max 30 [31]. Raw counts of mouse RefSeq genes were acquired with R package “Genomic Alignments” [32]. Differential gene expression analysis was performed with DESeq2 [33]. Gene Ontology (GO) and Kyoto Encyclopedia of Genes and Genomes (KEGG) enrichment analyses were performed using Clue GO based on significantly differentially expressed genes (false discovery rate < 0.05) from DESeq2 [1]. Enriched GO terms or KEGG pathways were visualized with Cytoscape V3 [34].

## 3. Results

### 3.1. Blastocyst Cell Number

**Figure 1:**
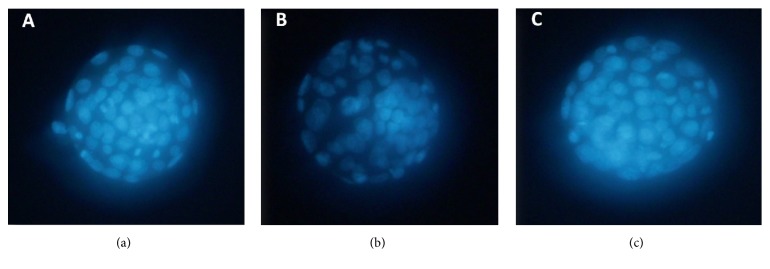
Fluorescence photomicrographs of blastocyst cells stained with Hoechst 33342. (a) Stained blastocyst cells in control group. (b) Stained blastocyst cells in model group. (c) Stained blastocyst cells in drug group.

In the control group (Figure 1(a)), the cells in blastocyst showed bright nuclear fluorescence. But in the model group (Figure 1(b)), blastocysts showed decreases in both nuclear fluorescence and the number of nuclei. In contrast to this, in the drug group (Figure 1(b)), both nuclear fluorescence and the number of nuclei in blastocysts increased compared to the model group. There were significant differences in the blastocyst cell number between the control group and the model group (*P* < 0.05) and between the model group and the drug group (*P* < 0.05) (Table 1).

### 3.2. Summary of Transcriptome Analysis

**Figure 2:**
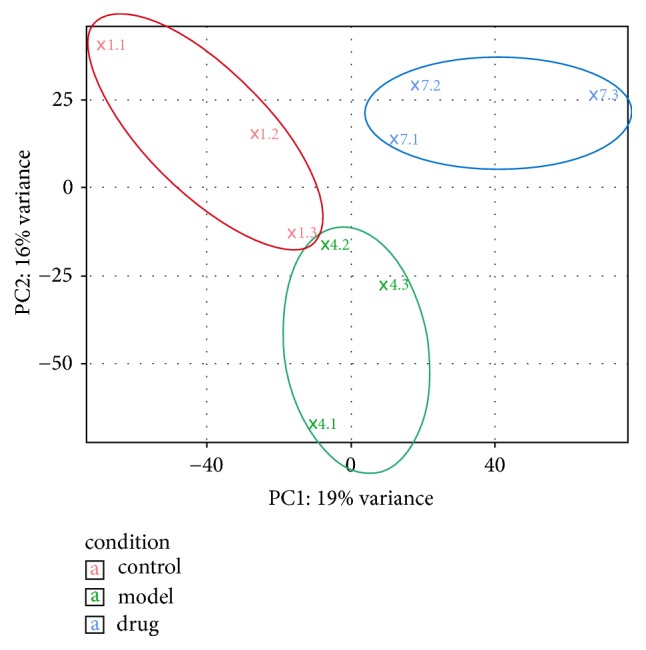
PCA of gene expression patterns for 2-cell stage embryos. Different colors identify different groups as indicated in the legend.

**Figure 3:**
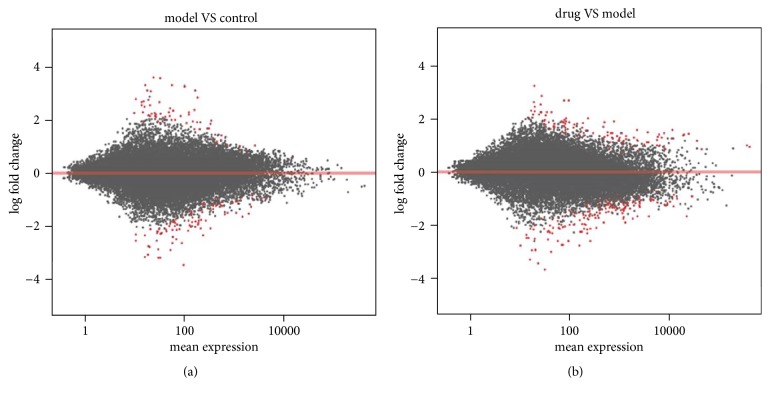
*MA*-plot of the log⁡2 fold changes over the mean of RNA-Seq read counts. The log⁡2 fold change for a particular comparison is plotted on *y*-axis and the average of the counts normalized by size factor is shown on *x*-axis. Each gene is represented with a dot, and genes with FDR < 0.05 are shown in red. (a) Comparison of model group with control group. (b) Comparison of drug group with model group.

Principal component analysis (PCA) was able to resolve and separate the three groups (Figure 2), indicating overall differences in the patterns of gene expression in these treatments. The *MA*-plot (“*M*” is for log ratios and “*A*” is for mean average) (Figure 3) demonstrates that most of the points were clustered tightly around the horizontal line, indicating that RNA-Seq data were of good quality. There were differently expressed genes between these three groups (red dots), indicating that expression of some genes was changed after the control group was loaded with high glucose (Figure 3(a)) and after the model group was treated with ZGW rat serum (Figure 3(b)). Furthermore, by comparing the global gene expression changes between model group and control group, we identified 71 upregulated and 100 downregulated genes, respectively. We then also compared the drug group to the model group, and 115 upregulated and 174 downregulated genes were detected (Table 2).

### 3.3. Pathway Analysis of Embryos Treated with High Glucose

**Figure 4:**
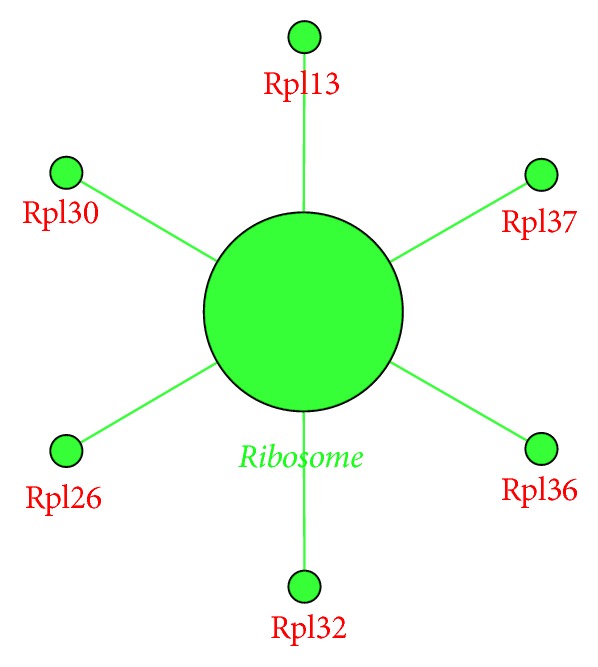
KEGG pathway (FDR < 0.05) enriched by the 171 differentially expressed genes in model group compared to control group. Green colored dots are downregulated. The big dot is a sign of the enriched pathway and the big green word is the name of the pathway. Small green dots show the genes that are downregulated, and the red words are the names of downregulated genes.

To identify the gene regulatory pathways potentially affected when embryos were treated with high glucose, two gene enrichment analyses were carried out for the differentially expressed genes between the model and control groups. For the Gene Ontology (GO) enrichment analysis (Table 3), we found 6 enriched biological process GO terms, including* “gluconeogenesis”* (GO: 0006094). For cellular components, we found* ribosome* (GO: 0022625, GO: 0015934, and GO: 0022626) and* mitochondrial respiratory chain* (GO: 0005746) as overrepresented terms. The* insulin-like growth factor binding* (GO: 0005520) was identified as the most significant overrepresented GO term in the molecular function category. Based on KEGG enrichment analysis, ribosome was identified as the only significantly overrepresented pathway with differentially expressed genes enriched in 2-cell stage of mouse embryo treated with high glucose (Figure 4).

### 3.4. Gene Expression Changes in High-Glucose Loaded Embryos Treated with ZGW Rat Serum

**Figure 5:**
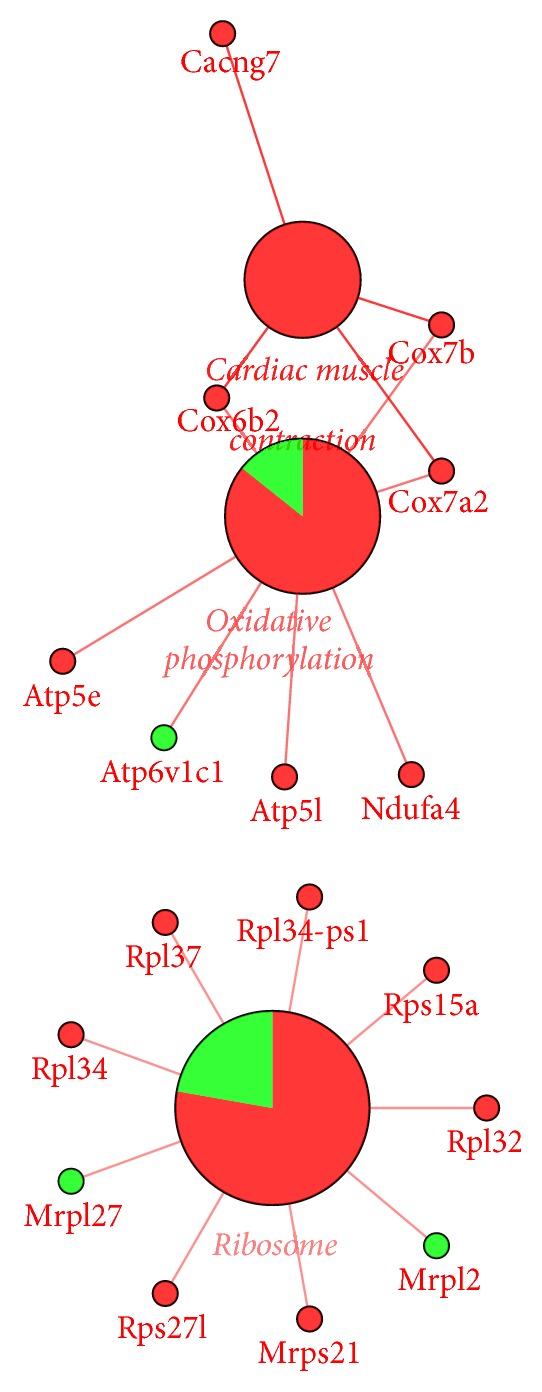
KEGG pathway (FDR < 0.05) enriched by the 289 differentially expressed genes in drug group compared to model group. Red means upregulated, while green means downregulated. Big dots represent the enriched pathways and big red words are the names of these pathways. Small red dots show the upregulated genes and the related red words are downregulated gene names. Small green dots show the downregulated genes and the related green words are downregulated gene names.

We used the same enrichment analysis to analyze the potential molecular pathways affected by ZGW in high-glucose loaded embryos. Based on differentially expressed genes in the drug group compared to the model group, 22 overrepresented GO terms were observed and 13 of them were in the molecular function category (Table 4). These terms included* hydrogen ion transmembrane transporter activity* (GO: 0015078),* cytochrome-c oxidase activity* (GO: 0004129),* heme-copper terminal oxidase activity *(GO: 0015002), and* oxidoreductase activity *(GO: 0016676, GO: 0016675). For cellular component terms, we found two main locations, the ribosome and mitochondria, with terms such as* ribosomal subunit* (GO: 0044391),* small nucleolar ribonucleoprotein complex *(GO: 0005732),* mitochondrial proton-transporting ATP synthase complex* (GO: 0005753), and* mitochondrial membrane part* (GO: 0044455). Results of KEGG enrichment analysis showed that genes in the ribosome pathway were upregulated, and some genes in the oxidative phosphorylation pathway were also upregulated by ZGW (Figure 5).

### 3.5. Identification of Potential Response Genes for ZGW

**Figure 6:**
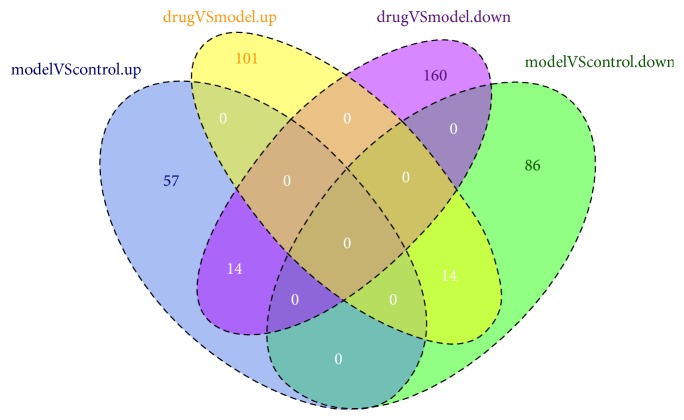
Venn diagram of pairwise gene expression changes between the three groups. Numbers in ovals represent different gene numbers between two groups.

**Figure 7:**
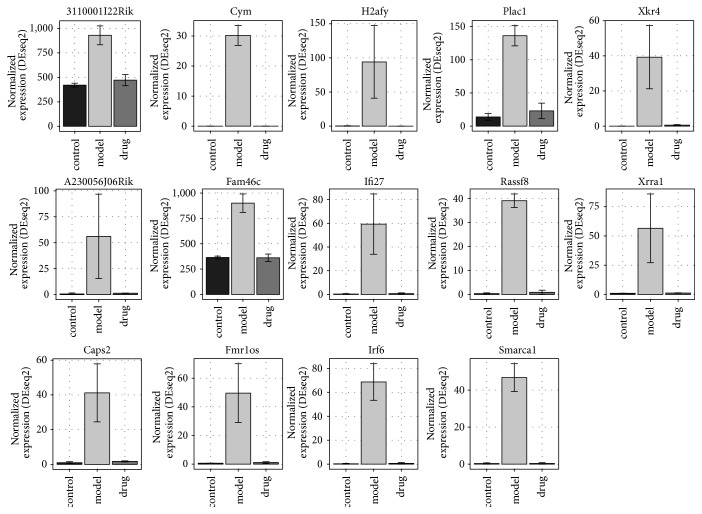
14 genes upregulated in model group and downregulated in drug group. Read counts normalized by library size according to DESeq2 are shown on *y*-axis.

**Figure 8:**
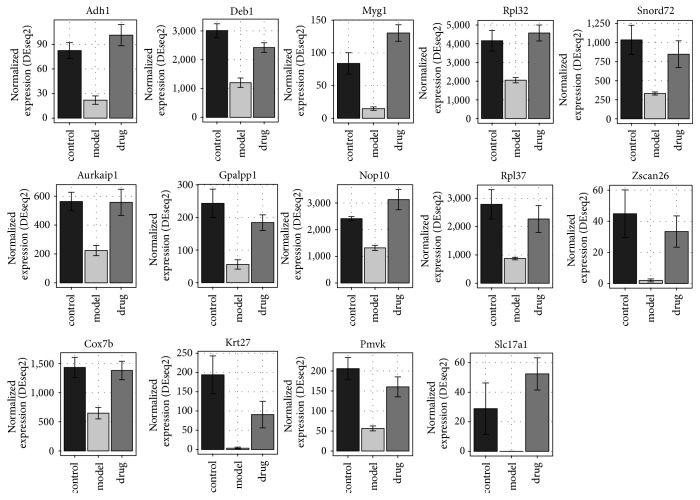
14 genes downregulated in model group and upregulated in drug group. Read counts normalized by library size according to DESeq2 are shown on *y*-axis.

Among the 71 upregulated genes in the model group compared to the control group, 14 genes were downregulated in embryos treated with ZGW (Figure 6). These genes were* Irf6, H2afy, Smarca1, Fmr1os, Ifi27, Fam46c, Cym, Caps2, Rassf8, Xkr4, Plac1, Xrra1, 3110001I22Rik, *and* A230056J06Rik* (Figure 7). No GO terms could be enriched based on these 14 genes. In the 100 downregulated genes from the model group compared to the control group, 14 genes were upregulated in embryos treated with ZGW (Figure 6). They were* Krt27, Deb1, Myg1, Pmvk, Rpl37, Snord72, Gpalpp1, Zscan26, Rpl32, Adh1, Slc17a1, Aurkaip1, Nop10, *and* Cox7b* (Figure 8). These 14 genes were enriched for GO terms highly related to mitochondrial energy metabolism (Table 5).

## 4. Discussion

### 4.1. Effects of High Glucose on Mice Embryo Development

Ample studies have shown that high glucose can affect embryos and block embryo development [35–37]. Research on the mechanism of this embryo development block has shown that high glucose can induce reactive oxygen species and cause damage to the embryo through oxidative stress [37, 38]. In this study, we found that high glucose downregulated several genes in the ribosome pathway (Figure 4), and three GO terms of* ribosome* have been enriched based on the differentially expressed genes between the model and control groups. Ribosome mediates protein synthesis and the downregulation of genes involved in the ribosome pathway in high-glucose loaded embryos indicated that protein synthesis might be affected. Furthermore, 2-cell stage is a key stage between maternal regulation and zygotic regulation in mouse [39]. Downregulation of ribosomal genes at this stage could affect the regulation of this transition.

We also found that genes involved in gluconeogenesis, including* crtc2*,* Ppara*, and* Sds*, were all downregulated in the model group. Molecular function of “insulin-like growth factor binding (GO:0005520)” was also affected by high glucose (Table 3). Insulin growth factors (IGFs) are important proteins in the regulation of embryo development, and a study showed that IGFs were main endocrine factors in the regulation of embryo development [40]; IGF-I has effects on metabolic regulation in embryos [41, 42]. The gluconeogenesis process is one of the pathways regulated by sugar in the uterus [40].

All these indicated that downregulating genes in ribosome pathway and affecting the sugar metabolism of mice embryo at 2-cell stage are two of the main causes of embryo developmental block induced by high glucose.

### 4.2. Efficacy of ZGW on High-Glucose Loaded Mice Embryo

Previous studies showed that ZGW rat serum could induce cell proliferation and differentiation [43, 44], inhibit cell apoptosis [45], and promote germ cell and embryo development [30, 46]. Furthermore, it has also been shown that the administration of ZGW to GDM rats has a preventive effect on the offspring's IGT caused by a high-fat and high-sugar diet [27]. In our study, we found that ZGW could upregulate several genes in the ribosome pathway, which were downregulated in embryos loaded with high glucose (Figure 5). The ribosomal subunit (GO: 0044391) was also one of the terms that been affected by ZGW (Table 4). In addition, among the 14 downregulated genes in the model group but upregulated in the drug group (Figure 8),* Rpl32* and* Rpl37* are both involved in the ribosome pathway.

Furthermore, we found that some pathways associated with energy metabolism were altered in high-glucose loaded embryos after treatment with ZGW. ZGW upregulated genes in the oxidative phosphorylation pathway (Figure 5), consistent with activate energy metabolism and sugar metabolism in embryos.* Oxidoreductase activity *(GO: 0016676 and GO: 0016675),* mitochondrial proton-transporting ATP synthase complex* (GO: 0005753), and* mitochondrial membrane part* (GO: 0044455) were also enriched based on the differentially expressed genes between the drug and model groups (Table 4). The GO annotation and coexpression network of 14 genes downregulated in the model group but upregulated by ZGW in the drug group (Table 5) showed that some of them were associated with the mitochondrial energy metabolism.

By these results, taken together with the result showing that ZGW prevented the embryo cell death caused by high glucose and promotes the blastocyst formation rate and total cell numbers in blastocysts, we conclude that ZGW may reverse the inhibition of the ribosome pathway and increase mitochondrial energy metabolism, which were inhibited by high glucose, and prevent the mouse embryo cell death caused by high glucose.

## 5. Conclusions

IGT can be effectively modeled using mouse embryos loaded with high glucose, providing an effective means to explore its pathogenesis and molecular mechanisms. By analyzing the transcriptome of this IGT model treated with ZGW, we found that high glucose might affect sugar metabolism and influence the mitochondrial function of mouse embryos at the 2-cell stage and that ZGW can counteract this by upregulating genes in the respiratory chain and oxidative phosphorylation. Furthermore, ZGW can prevent the embryo cell death caused by high glucose through upregulating genes inhibited by high glucose in ribosome pathway.

## Figures and Tables

**Table 1:** Effects of ZGW rat serum on blastocyst cell number.

Group	Blastocyst cell number (*n*)
Control	68.4^a^ ± 2.4
Model	57.2^a,b^ ± 1.6
Drug	63.2^b^ ± 2.2

Values are mean ± SEM. Means in column with different superscripts indicate significant differences (*P* < 0.05).

**Table 2:** Summary of differentially regulated gene numbers among the three groups (FDR < 0.05).

	Control	Model	Drug
Control	N/A	**100**	**N/A**
Model	*71*	N/A	**174**
Drug	*N/A*	*115*	N/A

Numbers in italic are upregulated genes, while those in bold are downregulated (DOCX).

**Table 3:** Summary of GO terms for differentially expressed genes between model and control groups.

Ontology source	GO ID	GO term	Term *P* value
Biological process	GO: 0034616	Response to laminar fluid shear stress	1.07*E* − 04
	GO: 0006525	Arginine metabolic process	4.12*E* − 04
	GO: 0034405	Response to fluid shear stress	2.18*E* − 03
	GO: 0021885	Forebrain cell migration	1.63*E* − 02
	GO: 0035914	Skeletal muscle cell differentiation	1.75*E* − 02
	GO: 0006094	Gluconeogenesis	1.81*E* − 02

Cellular component	GO: 0022625	Cytosolic large ribosomal subunit	1.19*E* − 05
	GO: 0015934	Large ribosomal subunit	1.53*E* − 04
	GO: 0022626	Sytosolic ribosome	2.36*E* − 04
	GO: 0031941	Filamentous actin	1.19*E* − 03
	GO: 0005881	Cytoplasmic microtubule	8.66*E* − 03
	GO: 0005746	Mitochondrial respiratory chain	1.18*E* − 02

Molecular function	GO: 0005520	Insulin-like growth factor binding	6.88*E* − 04
	GO: 0016879	Ligase activity, forming carbon-nitrogen bonds	7.94*E* − 03
	GO: 0033613	Activating transcription factor binding	9.60*E* − 03

15 GO terms are significantly enriched by the 171 changed genes after control group loaded by high glucose.

**Table 4:** Summary of GO terms for differentially expressed genes between drug and model groups.

Ontology source	GO ID	GO term	Term *P* value
Biological process	GO: 1902600	Hydrogen ion transmembrane transport	2.60*E* − 04
	GO: 0070646	Protein modification by small protein removal	6.99*E* − 04

Cellular component	GO: 0005732	Small nucleolar ribonucleoprotein complex	3.34*E* − 06
	GO: 0005685	U1 snRNP	1.47*E* − 03
	GO: 0044391	Ribosomal subunit	1.52*E* − 03
	GO: 0005753	Mitochondrial proton-transporting ATP synthase complex	1.70*E* − 03
	GO: 0045259	Proton-transporting ATP synthase complex	2.52*E* − 03
	GO: 0016469	Proton-transporting two-sector ATPase complex	2.57*E* − 03
	GO: 0044455	Mitochondrial membrane part	3.11*E* − 03

Molecular function	GO: 0015078	Hydrogen ion transmembrane transporter activity	1.90*E* − 04
	GO: 0004129	Cytochrome-c oxidase activity	4.00*E* − 04
	GO: 0015002	Heme-copper terminal oxidase activity	4.00*E* − 04
	GO: 0016676	Oxidoreductase activity, acting on a heme group of donors, oxygen as acceptor	4.00*E* − 04
	GO: 0008553	Hydrogen-exporting ATPase activity, phosphorylative mechanism	4.19*E* − 04
	GO: 0016675	Oxidoreductase activity, acting on a heme group of donors	4.55*E* − 04
	GO: 0030515	snoRNA binding	3.03*E* − 03
	GO: 0036442	Hydrogen-exporting ATPase activity	3.79*E* − 03
	GO: 0016830	Carbon-carbon lyase activity	3.95*E* − 03
	GO: 0016814	Hydrolase activity, acting on carbon-nitrogen (but not peptide) bonds, in cyclic amidines	4.66*E* − 03
	GO: 0000062	Fatty-acyl-CoA binding	6.17*E* − 03
	GO: 0019783	Ubiquitin-like protein-specific protease activity	6.26*E* − 03
	GO: 0019843	rRNA binding	3.21*E* − 02

22 GO terms are significantly enriched by the 289 changed genes after model group treated with ZGW rat serum.

**Table 5:** Summary of GO terms of 14 genes downregulated in model group and upregulated in drug group.

GO ID	Description	*q*-value
GO: 0005743	Mitochondrial inner membrane	1.92*E* − 06
GO: 0019866	Organelle inner membrane	2.07*E* − 06
GO: 0005746	Mitochondrial respiratory chain	2.19*E* − 05
GO: 0070469	Respiratory chain	2.19*E* − 05
GO: 0044455	Mitochondrial membrane part	6.72*E* − 05
GO: 1990204	Oxidoreductase complex	1.54*E* − 04
GO: 0030964	NADH dehydrogenase complex	4.25*E* − 04
GO: 0005747	Mitochondrial respiratory chain complex I	4.25*E* − 04
GO: 0045271	Respiratory chain complex I	4.25*E* − 04

9 GO terms are significantly enriched by the 14 reversed genes in drug group.

## Data Availability

All data are contained within the article. Raw data and material are available at https://www.ncbi.nlm.nih.gov/geo/query/acc.cgi?acc=GSE85477.

## Abbreviations

ZGW: * Zuo Gui Wan*
TCM: Traditional Chinese medicine
IGT: Impaired glucose tolerance
NGT: Normal glucose tolerance
DM: Diabetes mellitus
YGW: * You Gui Wan*
BZD: * Ba Zhen Tang*
FBG: Fasting blood glucose
2hBG: 2-hour blood glucose
FINS: Fasting serum insulin
APN: Leptin and adiponectin
GDM: Gestational Diabetes Mellitus
PMSG: Pregnant mare's serum gonadotropin
hCG: Human chorionic gonadotropin
PCA: Principal component analysis
GO: Gene Ontology enrichment analysis
KEGG: Kyoto Encyclopedia of Genes and Genomes.*⁡*
